# RAD6B Plays a Critical Role in Neuronal DNA Damage Response to Resist Neurodegeneration

**DOI:** 10.3389/fncel.2019.00392

**Published:** 2019-08-23

**Authors:** Zhao Guo, Yingxia Tian, Yingli Guo, Boya Li, Xiangwen Liu, Kun Xie, Yanfeng Song, Degui Wang

**Affiliations:** ^1^Department of Anatomy and Histology, School of Basic Medical Sciences, Lanzhou University, Lanzhou, China; ^2^Department of Internal Medicine, Gansu Provincial Academic Institute for Medical Research, Lanzhou, China

**Keywords:** RAD6B, aging, histone ubiquitination, DNA damage, neurodegeneration, senescence

## Abstract

RAD6 participates in DNA double-strand breaks (DSBs) repair by ubiquitinating histone H2B in mitotic cells. In terminally differentiated cells, however, the mechanisms of DNA damage repair are less well known. In this study, we investigate whether RAD6B is involved in DSBs repair in neurons and effects of RAD6B deficiency on neuronal survival. We compared neurons of RAD6B-deficient mice with those of littermate wild type (WT) mice and induced DNA damage by X-ray irradiation. We provide evidence that RAD6B is essential for neural DDR and RAD6B deficiency results in increased genomic instability and neurodegeneration. Moreover, higher levels of p53 and p21 are present in the brains of RAD6B-deficient mice, which may be responsible for neuronal senescence, and degeneration. In addition, behavioral experiments show that RAD6B-deficient mice exhibit marked learning and memory deficits. In conclusion, these findings suggest that RAD6B is critical for neural integrity and that the absence of RAD6B accelerates neurodegeneration in mice.

## Introduction

Biological cells are affected by various genotoxic stressors from endogenous and exogenous sources, and it is estimated that a typical mammalian cell undergoes ∼2 × 10^5^ lesions per day (Harper and Elledge, 2007; Ouyang et al., 2015; White and Vijg, 2016; Hegde et al., 2017). DNA DSBs are the most harmful to cells (Jackson and Bartek, 2009; Ouyang et al., 2015), which often lead to chromosomal rearrangements, senescence, tumorigenesis, or cell death (Lee and Mckinnon, 2007; White and Vijg, 2016). To avert those potentially devastating consequences, cells initiate highly evolved DNA damage response (DDR), and detect and repair damaged DNA. DSBs always occur when cells are exposed to genotoxic agents such as ionizing radiation and oxidative stress and are detected by two sensor proteins, the MRE11/RAD50/NBS1 (MRN) complex and the Ku70/Ku80 heterodimer (White and Vijg, 2016). Then ATM is activated and phosphorylates histone variant H2AX to form γ-H2AX and bind to the break sites. γ-H2AX also forms a binding site for MDC1, which recruits additional downstream repair factors, such as RNF8, 53BP1 and BRCA1, to gather at the sites and repair these breaks (Harper and Elledge, 2007; Marteijn et al., 2009; Liu et al., 2013; Ouyang et al., 2015). Based on different factors, including the cell type, cell-cycle stage and the severity of the damage, accurate repair leads to restoration of an intact double helix, while failed repair often cause apoptosis, and senescence or even tumor formation (Barzilai, 2010).

Protein ubiquitination is a series of complex cascade reactions including three enzymes: ubiquitin activating enzymes (E1s), ubiquitin conjugating enzymes (E2s), and ubiquitin ligases (E3s) (Walden et al., 2003; Chen et al., 2012). Along with other modifications such as methylation, acetylation and phosphorylation, ubiquitination plays important roles in many cellular processes (Kim et al., 2009), including the regulation of cell cycle, apoptosis, gene expression, transcription regulation, DNA damage repair, and protein degradation (Ciechanover, 1994; Biochem, 1998; Varshavsky, 2005; Chen et al., 2012). RAD6 is first identified as an important E2 in yeast and interacts with three separate E3s (Ubr1, Rad18, and Bre1) to participate in DDR. However, there are still some problems to be clarified for RAD6-mediated DNA repair involving its function with Bre1 in mono-ubiquitinating the histone H2B residue lysine 123. RAD6 mutation causes yeasts to be highly sensitive to ultraviolet light (Game and Chernikova, 2009; Chen et al., 2012). In addition, *Saccharomyces* mutants deficient in H2B mono-ubiquitination also shown irradiation-sensitive and abortive DNA damage repair, gene silencing, and cell-cycle checkpoints (Game and Chernikova, 2009; Chernikova et al., 2012). RAD6A and RAD6B are two homologs of yeast RAD6 and mice are non-viable if both homologs are absent (Chen et al., 2012).

While in the central nervous system, protected by the skull and blood-brain barrier, the most common threat to the neuronal genome comes from metabolic reactive oxygen species (ROS), which cause oxidative DNA damage, including single strand breaks (SSBs) (Madabhushi et al., 2014). However, under certain conditions, SSBs may be converted to DSBs, such as two SSBs that are in close proximity to each other (Madabhushi et al., 2014). And recent studies have shown that DNA DSBs are also formed in normal physiological/metabolic processes. On the other hand, the ability of neurons to repair damage decreases with aging. Thus, appropriate responses to DNA DSBs and accurate repair of broken DNA are required to maintain homeostasis and organismic survival (Lee and Mckinnon, 2007).

In recent years, there has been a continuous stream of evidences that mutations of DNA repair genes frequently cause severe defects in the central nervous system (Herrup et al., 2013) and that DNA damage is not only correlated with the aging process but also proposed to accelerate it (Barzilai and Mckinnon, 2013; Canugovi et al., 2013; Hegde et al., 2017). Furthermore, several neurodegenerative diseases, such as ataxia-telangiectasia (A-T), Alzheimer’s disease (AD), and Parkinson’s disease (PD), are closely related to DNA damage repair (Jackson and Bartek, 2009; Reynolds and Stewart, 2013). Our latest experiments have shown that loss of the ubiquitin ligase RNF8 leads to neurodegeneration in mice and DNA damage preceding dopamine neuron degeneration in PD mice (Ouyang et al., 2015; Wang et al., 2016). Bioinformatics analysis also indicates that 293T cells with RAD6B deficiency express higher levels of mRNA involving neurodegeneration. To further explore the effect of RAD6B deficiency on neurons, we focus on the function of RAD6B in neural DDR and phenotypes of neurons in RAD6B-deficient mice.

## Results

### RAD6B Is Essential for Neural DNA DSBs Repair

**FIGURE 1:**
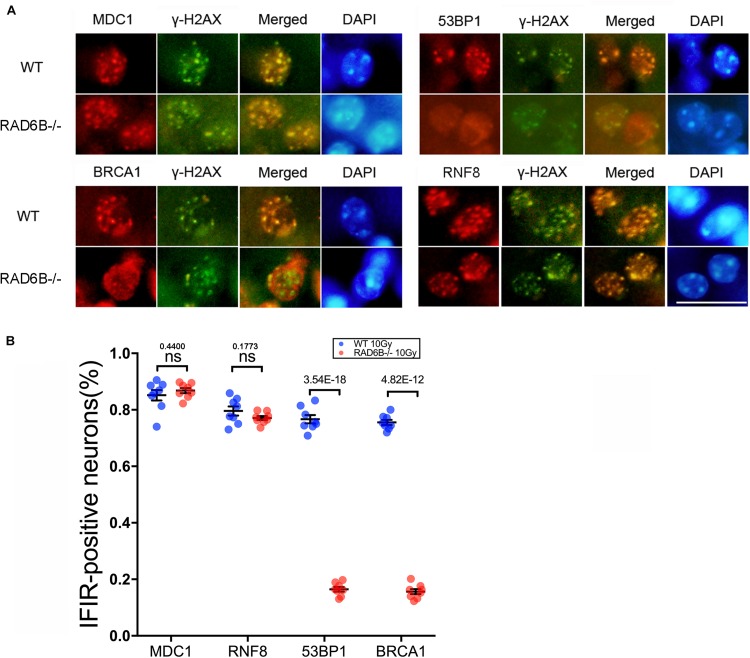
RAD6B is involved in DNA damage repair in neurons after IR. **(A)** RAD6B-deficient mice and control mice were simultaneously exposed to X-rays at a dose of 10 Gy. After 2 h of recovery, frozen sections of brain tissues were obtained and immunofluorescence was used to detect the formation of IRIF. Anti-MDC1, anti-53BP1, anti-BRCA1, and anti-RNF8 antibodies were applied separately for co-immunostaining with anti-γ-H2AX antibody. Scale bar = 20 μm. **(B)** The average percentages of IRIF-positive neurons were analyzed and are displayed with scatter diagrams. All values are presented as the mean ± SEM (*n* = 8). Student’s *t*-test.

To compare the repair processes for DSBs in the neurons of RAD6B-deficient mice with those in the neurons of WT mice, we observed the formation of ionizing radiation-induced nuclear foci (IRIF) at the broken sites after inducing damage by X-ray irradiation. γ-H2AX foci were used as a marker of DNA damage, while MDC1, 53BP1, RNF8, and BRCA1 were co-stained with γ-H2AX as repair factors. As shown in Figure 1, there were numerous 53BP1, BRCA1, RNF8, and MDC1 foci in the neurons of WT mice. In addition, most of them colocalized with the corresponding γ-H2AX foci, indicating that they were recruited to the broken sites after X-ray irradiation. By contrast, 53BP1 and BRCA1 foci were decreased dramatically in the neurons of RAD6B-deficient mice after X-ray irradiation, suggesting that RAD6B deficiency caused them to be recruited less to the break sites. However, in the neurons of RAD6B-deficient mice, almost the same proportions of MDC1 and RNF8 foci of were detected as in the neurons of WT mice, respectively (Figures 1A,B). These results suggest that RAD6B is involved in the repair process for DNA DSBs in neurons. Deficiency of RAD6B leads to incomplete recruitment of downstream repair factors, including 53BP1 and BRCA1, but the upstream proteins MDC1 and RNF8 are not affected.

### Loss of RAD6B Leads to Decreased H2B Ubiquitination

**FIGURE 2:**
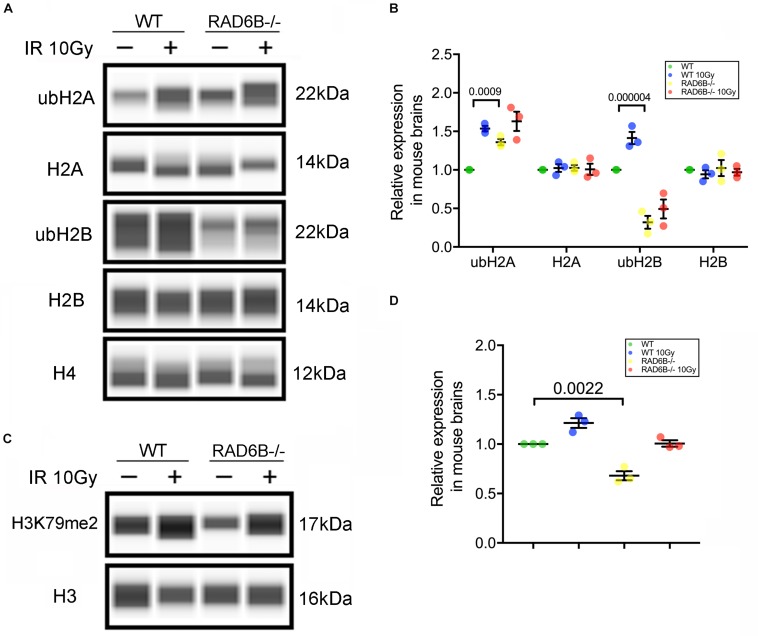
Loss of RAD6B causes decreased H2B ubiquitination. **(A)** RAD6B-deficient mice and control mice were treated with 10 Gy of X-rays or not. After recovery, histones were extracted from the brain in diluted hydrochloric acid, and the relative levels of ub-H2A, ub-H2B, H2A, H2B, and H4 were detected through western blotting. **(B)** The gray values of each group were measured, and the relative expression is displayed with a scatter diagram. All values are presented as the mean ± SEM (*n* = 3). Student’s *t*-test. **(C)** The relative levels of dimethylation of histone H3K79 were detected through western blotting. **(D)** The scatter diagram showing the relative expression of dimethylation of histone H3K79. All values are presented as the mean ± SEM (*n* = 3). Student’s *t*-test.

Post-translational modifications (PTMs) of histones are important regulators of the structure of chromosomes. We next focused on the substrate histones of RAD6B ubiquitination in neurons and measured the ubiquitination levels of the histones H2A and H2B. As shown in Figures 2A,B, knockout of RAD6B leads to significant reduction in the ubiquitination of H2B compared with the WT mouse brains. No evident increase was detected in the expression of ubH2B in RAD6B-deficient mouse brains, while ubH2B in the WT mouse brains increased obviously after X-ray irradiation. However, ubH2A in the brains of WT mice and RAD6B-deficient mice both increased after IR, and higher level of ubH2A was observed in the brains of RAD6B-deficient mice. The relative expression levels of H2A and H2B did not show clear changes between groups (Figures 2A,B). Consistent with ubH2B, the level of H3K79me2 in RAD6B-deficient mouse brains decreased significantly (Figures 2C,D).

### RAD6B Deficiency Leads to Increased Genomic Instability and Neurodegeneration

**FIGURE 3:**
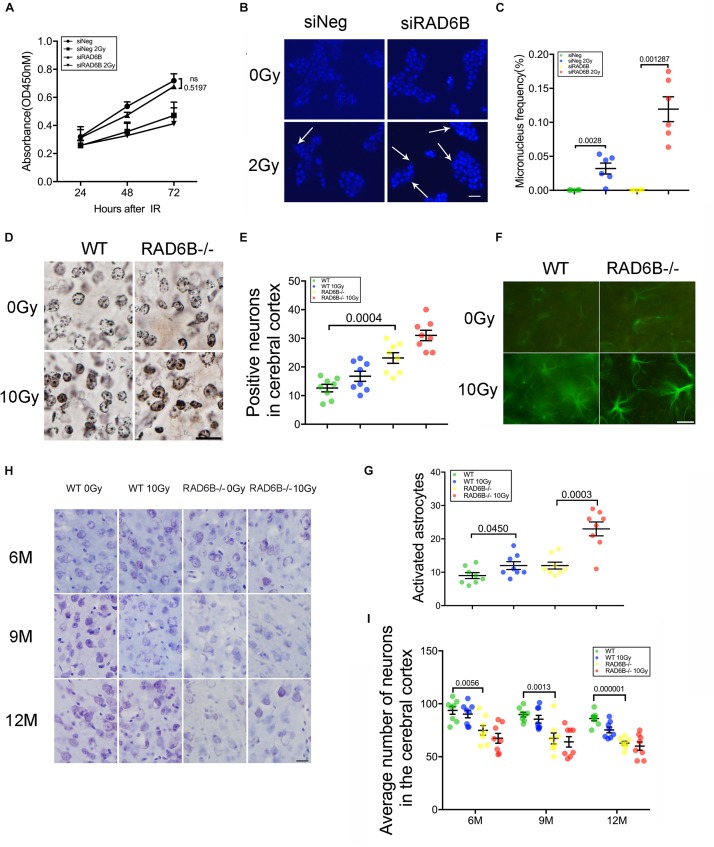
Deficiency of RAD6B leads to neurodegeneration and loss of neurons. **(A)** Proliferation of siNeg or siRAD6B-treated RPE1 cells after 2Gy of X-ray irradiation or not. All values are presented as the mean ± SEM (*n* = 3). Two-way ANOVA. **(B)** Photomicrographs of siNeg or siRAD6B-treated RPE1 cells scored in the MN test, the arrow indicating the representative MN. Scale bar = 50 μm. **(C)** The frequency of MN formation in RPE1 cells, the data are presented as means ± SEM (*n* = 6). Student’s *t*-test. **(D)** Plot of neurons in silver-stained slices from 12-month-old mice. Scale bar = 20 μm. **(E)** Scatter diagram displaying the average density of abnormal neurons in the cerebral cortex of 12-month-old mice. All values are presented as the mean ± SEM (*n* = 8). Student’s *t*-test. **(F)** Two hours after X-ray irradiation, the brain slices of 6-month-old mice underwent immunofluorescence staining of GFAP to show astrocytes. Scale bar = 20 μm. **(G)** A scatter diagram showing the relative activation of astrocytes in the brains of mice in each group after 2 h of recovery. All values are presented as the mean ± SEM (*n* = 8). Student’s *t*-test. **(H)** CV staining of slices from mice of different ages; the mice were treated with X-rays and allowed to recover for days or were not treated with X-rays. Scale bar = 20 μm. **(I)** Scatter diagrams indicating the average number of neurons in each group at different ages. All values are presented as the mean ± SEM (*n* = 8). Student’s *t*-test.

While knocking down RAD6B did not significantly influence cell proliferation compared with siNeg treated cells within 72 h, cell growth was obviously inhibited with 2Gy of X-ray treatment (Figure 3A). To determine the effect of RAD6B deficiency on neuronal genomic integrity, we evaluated the formation of MN. A total of 1000 binucleated cells in six separate experiments for each group were scored to evaluate the frequency of MN induction and cells contained at least one MN were counted as MN-positive. Shin Koyama reported that there was a threshold of dose of X-rays for MN induction and no significant difference at doses lower than 0.05Gy was detected compared with the control. Interestingly, a small amount of MN was also detected in siRAD6B-treated RPE1 cells without X-ray irradiation. However, in the case of X-ray treatment, a large amount of MN was produced, about four folds as much as that of siNeg treated cells (Figures 3B,C). Generally, MN originate from chromosome fragments or whole chromosomes lagging behind during anaphase of mitosis. Therefore, these results suggest that RAD6B deficiency causes increase of genomic instability in neurons due to the defect of DSBs repair.

Analysis of the nuclei and neurites in neurons of the RAD6B-deficient mice with silver staining revealed that more nuclei in RAD6B-deficient mice became irregular and deeply stained, while neurons in the WT mice had relatively regular morphology and included only a few deeply stained cells. X-ray irradiation increased the staining of some nuclei, but the neurons in RAD6B-deficient mice were more pronounced (Figures 3D,E). More degenerated neurites (tangles, thickening, and deeper staining) were also observed in the brains of RAD6B-deficient mice (Supplementary Figure S2C).

Astrocytes, as the most important supporting cells in the central nervous system, become larger and grow longer protrusions with more branches when activated. Furthermore, upregulation of glial fibrillary acidic protein (GFAP) in activated astrocytes indicates neuronal injury and degeneration. We measured GFAP and found that there were more astrocytes activated in RAD6B-deficient mice than in WT mice. X-ray irradiation also increased the activation of astrocytes in the brains of RAD6B-deficient mice and WT mice, and RAD6B-deficient mice showed significantly more astrocytic activation after X-ray irradiation (Figures 3F,G). In addition, there were more degenerated neurons surrounded by microglial cells in RAD6B-deficient mice (Supplementary Figures S2D,E).

Analysis of immunohistochemistry with anti-NeuN revealed that the number of neurons in the RAD6B-deficient mice was significantly reduced compared with homologous 12-month-old WT mice; the RAD6B mutants had approximately 3/4 as many neurons as the WT mice. In addition, RAD6B-deficient mice lost more neurons than WT mice after X-ray irradiation (Supplementary Figures S2A,B). We further performed CV staining to investigate neuronal loss in those knockout mice over time. The loss of pyramidal neurons resulting from RAD6B deficiency gradually increased, and those mice lost more neurons than homologous WT mice from 6 to 12 months of age (Figures 3H,I). Above all, changes in brain slices demonstrate that RAD6B deficiency results in a reduction in the number of neurons.

**FIGURE 4:**
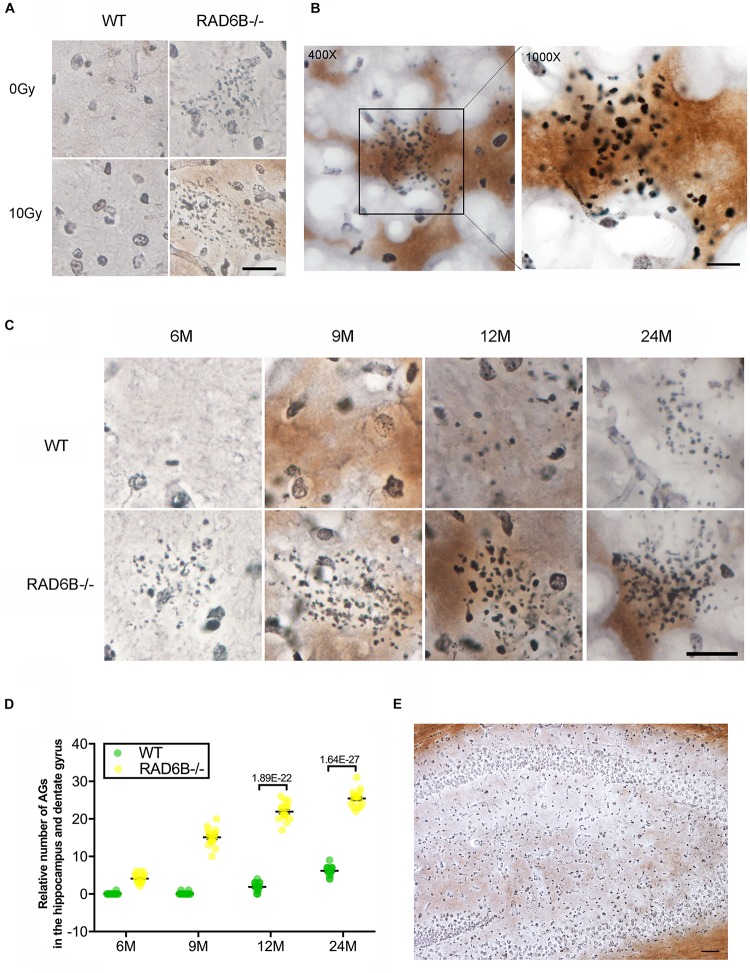
RAD6B deficiency leads to abnormal accumulation of argyrophilic grains (AGs) in the hippocampus and dentate gyrus. **(A)** Brain slices were stained with silver, showing abnormal accumulation of AGs in the hippocampus. RAD6B-deficient mice and control mice were treated with 10 Gy of X-rays or left untreated. Scale bar = 20 μm. **(B)** A representative image shows the abnormal accumulation of AGs in the hippocampus and a magnified detail of the image. **(C)** Aggregation of AGs in hippocampus of WT mice and RAD6B-deficient mice at different ages. Scale bar = 10 μm. **(D)** The scatter diagram shows the relative number of AGs abnormally aggregated in the hippocampus of mice of different ages. All values are presented as the mean ± SEM (*n* = 18). Student’s *t*-test. **(E)** The silver-stained brain slices show that AGs are widely distributed in the hippocampus and dentate gyrus. Scale bar = 100 μm.

Argyrophilic grain disease, characterized by the presence of argyrophilic grains, is a progressive degenerative disorder of neurons and becomes increasingly prevalent with advancing age (Braak and Braak, 1998; Yoshida et al., 2017). Unexpectedly, we found a large number of argyrophilic grains (AGs) in the hippocampus and dentate gyrus in 9-month-old RAD6B-deficient mice (Figures 4A,B). Moreover, there was no significant change in the number of AGs after X-ray irradiation. After a further study of age-related changes in AGs, we noticed that numerous AG deposits were present in the hippocampus and dentate gyrus in 6-month-old RAD6B-deficient mice. However, there were only sporadic deposits in brain slices from 12-month-old WT mice. RAD6B-deficient mice had more AG aggregates than their WT littermates at different ages (Figures 4C–E). These results further suggest that RAD6B deficiency accelerates neuronal senescence.

### RAD6B Deficiency Results in Neuronal Senescence

**FIGURE 5:**
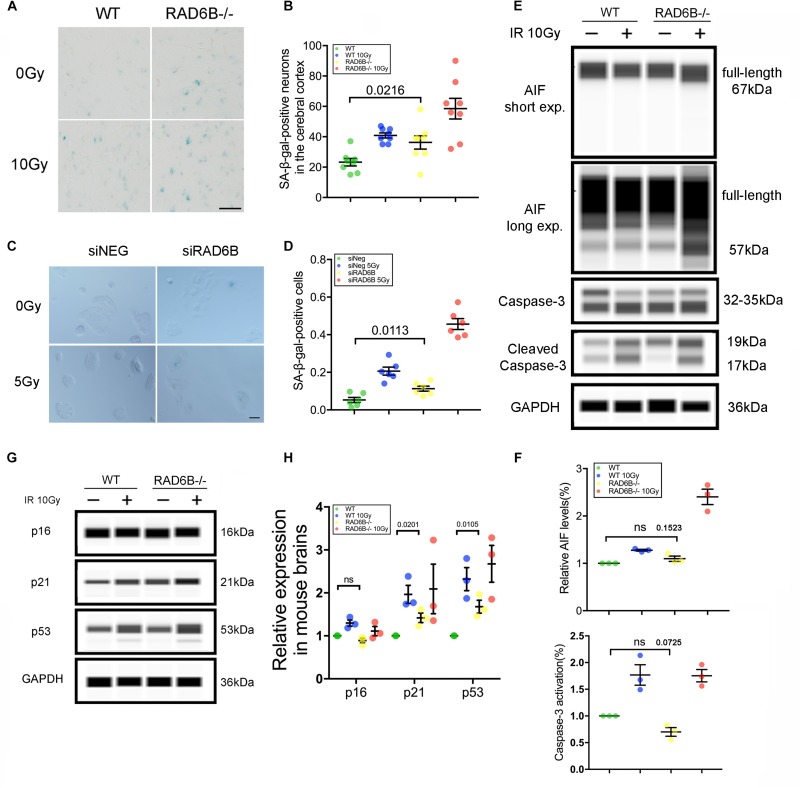
RAD6B deficiency leads to increased neuronal senescence. **(A)** β-Galactosidase staining of slices from 12-month-old mice; the mice were either treated with X-rays and allowed to recover for 10 days or not treated with X-rays. Scale bar = 20 μm. **(B)** The scatter diagram showing the average number of senescent neurons in the cortex. All values are presented as the mean ± SEM (*n* = 8). Student’s *t*-test. **(C)** Photomicrographs of β-Galactosidase staining of siNeg or siRAD6B-treated RPE1 cells. Scale bar = 50 μm. **(D)** The bar chart displaying the percentage of senescent RPE1 cells, the data are presented as means ± SEM (*n* = 6). Student’s *t*-test. **(E)** The representative blot presenting caspase-3 and AIF levels analyzed through simple western. **(F)** A scatter diagram displaying the relative levels of cleaved caspase-3 and mature form of AIF (57 kDa) in each group. All values are presented as the mean ± SEM (*n* = 3). Student’s *t*-test. **(G)** RAD6B-deficient mice and control mice, treated with 10 Gy of X-rays, or not. After a 2 h recovery period, the total protein of the brain was extracted, and a western blot was performed with the following primary antibodies: p16, p53, p21, and GAPDH. **(H)** The gray values of each group were measured, and the scatter diagram indicates the relative levels of these proteins. All values are presented as the mean ± SEM (*n* = 3). Student’s *t*-test.

DNA damage repair defects lead to accumulation of damages, resulting in apoptosis and senescence, which are widely accepted as the major mechanisms inhibiting tumor formation mediated by p53 (Campisi, 2005; Li et al., 2012). To evaluate the effects of RAD6B deficiency on neuron survival, we compared the senescence of neurons in the four groups of mice. Senescent cells are usually larger in size and express β-galactosidase with high enzyme activity at pH 6.0. The senescent neurons in the RAD6B-deficient mice were slightly more numerous than those in the WT mice, and greater numbers of senescent neurons were observed after X-ray irradiation in 12-month-old mice (Figures 5A,B). Similarly, RAD6B silencing also causes RPE1 cells to show increased activity of SA-β-gal as more senescent cells were detected compared to control cells 7 days after X-ray treatment (Figures 5C,D). Next, we compared the levels of activated caspase-3 and mature form of AIF in WT mice and RAD6B-deficient mice to assess apoptosis, but no significant difference was detected (Figures 5E,F). These results suggest that inhibition of RAD6B induces defects in DNA damage repair and that neurons are more susceptible to senescence.

### Activation of P53 and P21 Contributes to the Senescence of RAD6B Deficient Neurons

Failure of DNA damage repair leads to senescence, usually through two signaling pathways: the p53–p21 pathway and the p16–Rb pathway (Sherr and McCormick, 2002; Campisi, 2005; Cao and Li, 2015; Gil and Withers, 2016). To investigate the causes of neuronal senescence in RAD6B-deficient mice, we examined key factors in these two pathways. As shown in Figures 5G,H, the levels of p21 increased in both groups after X-ray irradiation. Meanwhile, in the absence of X-ray irradiation, the levels of p21 in RAD6B-deficient mice also increased significantly. In addition, changes in the expression of p53 protein were similar to the changes in p21. However, p53 had relative markedly higher expression than p21 in RAD6B-deficient mouse brains. The levels of p16 did not vary obviously between groups (Figures 5G,H). Those results suggest that deficiency of RAD6B leads to increased expression of p53 and p21, which may contribute to neural senescence in RAD6B-deficient mice.

### RAD6B Deficient Mice Exhibit Learning and Memory Impairment

**FIGURE 6:**
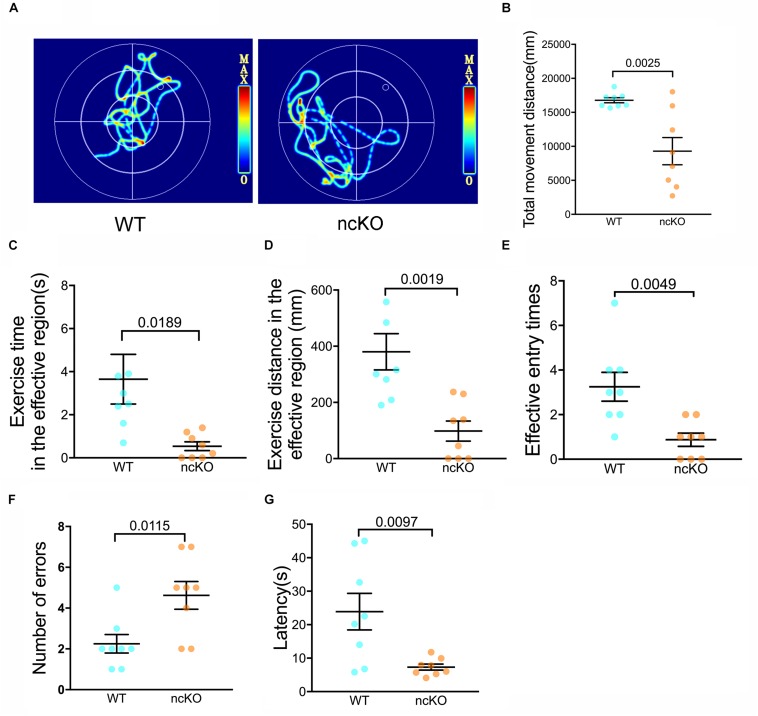
RAD6B-deficient mice exhibit weight loss accompanied by learning and memory deficits. **(A)** Motion paths of RAD6B ncKO mice and normal mice in the Morris water maze **(B–E)**. The scatter diagrams show total movement distance, time and distance in the target region and the frequency with which the two genotypes entered the target region in the Morris water maze. All values are presented as the mean ± SEM (*n* = 8). Student’s *t*-test. **(F,G)** The number of errors and the latency of the first error on the passive avoidance response test are summarized and displayed in scatter diagrams. All values are presented as the mean ± SEM (*n* = 8). Student’s *t*-test.

As the command center of the nervous system, the brain controls a number of functions, including learning and memory, motor control, homeostasis, information processing, and perception (Rulten and Caldecott, 2013). Interesting, we noticed that the learning and memory abilities of RAD6B-deficient mice were significantly lower than those of WT mice. To identify whether these changes were caused by RAD6B systemic knockout, we used RAD6B neuron-conditional knockout mice (ncKO) and their WT littermate controls to evaluate the differences in motor performance and learning and memory through avoidance experiments and Morris water maze. RAD6B ncKO mice looked more inactive and exhibited slow behavior and decreased movement, and the two genotypes showed apparent difference in motor ability (Figures 6A,B). In addition, RAD6B ncKO mice were smaller in size than the WT mice and weighed significantly less (Supplementary Figures S1C,D).

We subjected both genotypes to a spatial exploration experiment to evaluate their spatial learning and memory ability in the Morris water maze. The WT mice were more likely to enter the target region to find the escape platform, with shorter latency to enter the target region, a longer total duration of time spent there, and more entries into the region (Figures 6C–E).

In the passive avoidance response test, RAD6B ncKO mice made twice as many errors as WT mice (Figure 6F). In addition, their error latency was significantly shorter than that of WT mice (Figure 6G). These results indicate that the learning and memory abilities of RAD6B-deficient mice are lower than those of WT mice during the early learning process.

## Discussion

Maintaining genetic stability, especially in the central nervous system, is vital to individual survival and reproduction. Most neurons in human beings are developed within 3 years and survive for the individual’s lifetime (Hegde et al., 2017). As DNA DSBs usually threaten cell survival, eukaryotic organisms will mainly repair the injury in two conservative ways: homologous recombination (HR) and non-homologous end joining (NHEJ) (Escribano-Díaz et al., 2013; Iyama, 2013). NHEJ can occur at any stage of the cell cycle, although it is not as precise as HR (Iyama, 2013; Rulten and Caldecott, 2013; Ouyang et al., 2015). Since neurons are terminally differentiated cells and cannot re-enter the cell cycle, there is no template for homologous recombination repair. NHEJ is the primary mechanism involved in neuronal DDR 4–6 h after X-ray irradiation and repairs 80–85% of DNA DSBs (Löbrich et al., 2010; Jeggo et al., 2011; Reynolds and Stewart, 2013; Ouyang et al., 2015). However, the mechanisms underlying this repair are less well known in terminally differentiated cells.

In response to DNA DSBs, the MRN complex and KU proteins will sense this fragmentation damage and rapidly recruit and activate downstream ATM and DNA-PKcs, respectively (Assenmacher and Hopfner, 2004; Jackson, 2005; Bartkova et al., 2008). ATM is phosphorylated at Ser1981 and binds to the break sites, where it assembles the histone variant H2AX and phosphorylates it at Ser139 (Lavin, 2007; Marteijn et al., 2009; Rulten and Caldecott, 2013). Subsequently, MDC1 recognizes and binds to γ-H2AX via the BRCT domains and recruits downstream two important cell cycle checkpoint mediators: 53BP1 and BRCA1, both of which are also involved in many aspects of DNA repair. While 53BP1 mainly acts through its effector Rif1 to promote NHEJ, BRCA1 facilitates homologous recombination after resection of the broken ends (Bunting et al., 2010; Escribano-Díaz et al., 2013). Interestingly, deficiency of RAD6B caused mice to form fewer 53BP1 and BRCA1 foci in neurons with X-ray treatment, while the underlying mechanism needs further study. Previous studies reported that MDC1 recruits RNF8 at damage sites through the interaction between RNF8 and UBC13, which would ubiquitinate H1 and initiate subsequent ubiquitination events, including ubiquitylation of H2A-type histones mediated by RNF168 (Marteijn et al., 2009; Fradet-Turcotte et al., 2013; Thorslund et al., 2015; Mandemaker et al., 2017). BRE1 facilitates mono-ubiquitination of H2B by RAD6 through its ring finger domain. In addition, ubH2B is essential for the dimethylation and trimethylation of histones H3K4 and H3K79 (Ng et al., 2002; Kim et al., 2009; Chen et al., 2012; Kleiner et al., 2015; Van Oss et al., 2016), which are required for activation of chromatin, remodeling, and activation of transcription of corresponding genes. Our results demonstrated that the ubiquitination level of H2B showed significant difference between RAD6B-deficient mouse brains and WT mouse brains, resulting in subsequent repair failures in neurons.

The developing and mature central nervous system responds differently to DNA damage, with the former tending to apoptosis, while the consequences of latter is less known (Lee and Mckinnon, 2007). Duchaud, E. et al. reported that mutations in ATM gene result in increased sensitivity of cells to ionizing radiation, however, they show less apoptosis than normal cells after irradiation (Roos and Kaina, 2006). Similarly, in this study, we noticed a remarkable increase in genomic instability in RAD6B deficient neurons, but no significant apoptosis compared with neurons from WT mice. Unrepaired DBSs caused by NHEJ deficiency have been reported to accelerate aging in mice and analysis of DSBR during aging revealed that cells from older mice contained more DSB than those from younger mice (Reynolds and Stewart, 2013; Rulten and Caldecott, 2013; Madabhushi et al., 2014). In addition, Approximately 20–40% of the cerebral cortex and hippocampus region in the brains of older C57BL/6 mice has been observed to have severe DNA damage, heterochromatin formation, and senescence-associated β-galactosidase activation (Jurk et al., 2012). Jurk et al. (2012) reported that neurons develop a p21-dependent senescence-like phenotype mediated by failed DNA damage response. Western blot showed elevated expression of p53 and p21 which may stimulate subsequent responses and cause senescence. And slices from RAD6B-deficient mice exhibit more deeply stained nuclei and increased activation of astrocytes compared to those from WT mice, suggesting more unrepaired lesions in those neurons and more neurons undergoing neurodegeneration associated with aging. Further, the loss of neurons was more severe in RAD6B-deficient mice than in WT mice, which corresponded to the decrease in ubiquitination of H2B and an increase in p53 and p21 expression. In combination with these results, the absence of RAD6B was responsible for the failure of repair, leading to neuronal senescence, and neurodegeneration.

As the mice grew, we observed physical and behavioral differences between mice; the RAD6B-deficient mice were lighter in body weight than the WT mice and appeared less active than the WT mice. We further explored the changes in their learning and memory through behavioral experiments. RAD6B-deficient mice showed reduced learning and memory ability in behavioral tests, indicating impairment in learning, and memory. In combination with neuronal changes in the mutant mice, we suggest that neurodegeneration may lead to these behavioral differences between RAD6B-deficient and wild-type mice.

Collectively, our data indicate that, in the central nervous system, RAD6B is essential for DDR and RAD6B deficiency causes defects in DNA DSBs repair and increased genome instability, which accelerates neuronal senescence and leads to neurodegeneration.

## Materials and Methods

### Generation of RAD6B KO Mice and X-Ray Irradiation

RAD6B knockout C57BL/6 embryonic stem (ES) cells were generated by the knockout mouse project (KOMP) repository (UC Davis). The ES cells contained an allele [Ube2btm1 (KOMP) Vlcg] with an ablating deletion of the RAD6B gene, inserted in place of the WT allele by an expression-selection cassette (VelociGene KOMP definitive null allele design). The ES cells were implanted in the uteri of mice to produce transgenic mice. RAD6B knockout mice were obtained by crossing the mice with lines containing CMV-cre for systemic knockout and Nes-cre for neuron-conditional knockout (Supplementary Figures S1A,B). All mice were feed and bred in an SPF laboratory at the animal center of Lanzhou University. We randomly selected RAD6B−/−, mice and homologous WT mice and divided them into four groups [WT, WT IR (with X-ray irradiation treatment), RAD6B−/− and RAD6B−/− IR]; each group contained 30 mice. X-ray irradiation was performed with an X-ray biological irradiation system (X-RAD 225 Cx, Pxinc). All experiments involved in this study were approved by the ethics committee of Lanzhou University.

### Tissues Sampling and Frozen Sections

Mice from each group were anesthetized with isoflurane, then perfused and fixed with 4% neutral formaldehyde. The brains were totally dissected and soaked in 20% and 30% sucrose solution for gradient dehydration. All the sections were cut at a thickness of 25 μm on a freezing microtome.

### Cells Culture

The immortalized mouse hippocampal neuronal RPE1 cells were purchased from ATCC and cultured in DMEM with high glucose supplemented with 10% fetal bovine serum (Gibco, Frederick, MD, United States) at 37°C in a humidified incubator with 5% CO_2_ and 95% air. While for SA-β-Gal staining and micronucleus test, cells were seeded into coverslip in Petri dishes at a density of 1 × 10^5^ cells per mL and grown for days until 70–80% confluent.

### Silencing of RAD6B Gene

RPE1 cells between passages 5 and 15 were infected with lentivirus particles (GeneChem Co., Shanghai, China) at a multiplicity of infection (MOI) of approximately 50 to generate siRNA against RAD6B (siRAD6B) or non -targeting siRNA (siNeg) [siRAD6B: 5′ ACCAGAAGGGAC ACCCTTTGAAGATG3′; siNeg: negative control (GeneChem)]. 12 h after infection, the medium was replaced by complete medium, and cultured for subsequent days. The RNA interference efficiency was confirmed by western blot.

### Immunofluorescence Staining

Brain slices from 6-month-old WT IR and RAD6B−/− IR mice were subjected to immunofluorescence staining as described by Liu et al. (2013). The primary antibodies used were anti-γ-H2AX (ab26350, Abcam; 1:200), anti-GFAP (BA0056, BOSTER; 1:150), anti-BRCA1 (ab26350, Abcam; 1:200), anti-53BP1 (ab36823, Abcam; 1:200), anti-MDC1 (ab11169, Abcam; 1:200), and anti-RNF8 (ab26350, Abcam; 1:200). After the nuclei were stained with DAPI, photomicrographs were captured with an olympus fluorescence microscope (BX53).

### Western Blot Assay

Brain tissues from 6-month-old mice were frozen quickly in liquid nitrogen, after which histones, and total protein were extracted according to the standard methods provided by Abcam. The concentration of each protein of interest was measured with an ultra-microspectrophotometer (DS-11 FX, DeNovix). We performed automated western blots with Simple Western according to the manufacturer’s instructions. And histones were detected with primary antibodies against H1.2 (19649, Proteintech; 1:500), ub-H2A (clone E6C5, Millipore; 1:500), H2A (ab36823, Abcam; 1:200), ub-H2B (clone 56, Millipore; 1:500), H2B (15857, Proteintech; 1:1000) and H4 (16047, Proteintech; 1:2000), while total protein was reacted with antibodies against p21 (ab109199, Abcam; 1:1000), p16 (ab108349, Abcam; 1:1000), p53 (10442-1-AP, Proteintech; 1:500), AIF (17984-1-AP, Proteintech; 1:500), caspase-3 (19677-1-AP, Proteintech; 1:500), and GAPDH (10494-1-AP, Proteintech; 1:2000).

### Cell Proliferation

RPE1 cells were seeded at 1000 cells/well in 96-well dishes. After having been incubated for 12 h, cells were irradiated at dose of 2Gy or not. Following 24, 48, and 72 h of incubation, cell proliferation was measured by CCK-8 assay.

### Micronucleus Test

Following irradiation at a dose of 2Gy, cells were washed three times with phosphate buffer saline (PBS) and continued to culture in DMEM containing cytochalasin B at a concentration of 3 μg/ml for 16h to prevent cell division. After incubation, cells were washed and fixed with 10% neutral formaldehyde for at least 30 min. The cells were washed gently with PBS and stained with DAPI, and photomicrographs were captured with an BX53.

### Cresyl Violet (CV) Staining

After X-ray irradiation at a dose of 10Gy and days of recovery, slices from 6-, 9-, and 12-month-old mice were stained with 0.5% CV for 15 min and differentiated in 0.25% glacial acetic acid ethanol solution for 15 s, until the slices turned light blue. The slices were dehydrated with graded ethanol, cleared in xylene and covered with neutral balsam.

### Silver Staining

Brain slices from 12-month-old mice were incubated in 20% silver nitrate for 30 min at 37°C. After being washed 3 times in 0.01 M PBS, they were reduced in 10% neutral formaldehyde for 3 min, until they had turned brown. Following incubation in silver ammonia solution for 5 min at 37°C, slices were placed in 10% neutral formaldehyde for 40 s and toned with 0.2% gold chloride solution. Finally, the slices were rinsed in 5% sodium thiosulfate solution for 2 min. After being washed adequately with distilled water, all slices were dehydrated and covered with neutral gum.

### SA-β-GAL Staining

Senescent cells were stained with a Senescence β-Galactosidase Staining Kit (C0602, Beyotime) according to the standard procedures. The slices were fixed at room temperature for 20 min. After being washed three times, each slice was stained with the dyeing solution and incubated at 37°C overnight. The positive cells were observed by microscope after routine dehydration and mounting. Detection of senescence-associated β-galactosidase in RPE1 cells was also performed following the instructions.

### Behavioral Tests

RAD6B ncKO mice were subjected to the Morris water maze and a passive avoidance response test. For the Morris water maze, all mice were trained for 5 min on 7 consecutive days and assessed on the eighth day. The escape latency, total distance and time spent in the effective region were recorded automatically by a monitoring system (WMT-100, TECHMAN SOFT).

Passive avoidance response was performed with a video analysis system (PAT-8, TECHMAN SOFT). After the mice were allowed to move freely between the light chamber and the dark chamber for 3 min, the metal grids of the dark chamber were electrified with a voltage of 24 volts for 5 min. Prior to the formal test, all mice underwent two consecutive days of regular exposure and training. The Error counts and latency were recorded every 3 s.

## Data Availability

All datasets generated for this study are included in the manuscript and/or the Supplementary Files.

## Ethics Statement

All experiments involved in this study were approved by the ethics committee of Lanzhou University.

## Author Contributions

ZG and DW initiated the project and wrote the manuscript. ZG, YS, YT, and YG designed the experiments. ZG, YS, YT, YG, and BL performed the experiments. XL, YT, YG, and BL analyzed the data. KX, YG, and DW constructed the figures.

## Conflict of Interest Statement

The authors declare that the research was conducted in the absence of any commercial or financial relationships that could be construed as a potential conflict of interest.

## Supplementary Material

FIGURE S1 Implementation of RAD6B-deficient mice. **(A)** Editing the RAD6B gene through the Cre-loxP recombination system. CMV-cre and Nes-cre were used to produce systemic knockout mice and ncKO mice, respectively. **(B)** Identification of the genotypes of some offspring mice obtained from RAD6B heterozygous mice. Primers were designed for genome amplification. **(C)** Physical comparison of 4-month-old WT mice and RAD6B ncKO mice. **(D)** The scatter diagram shows weight differences between 18-month-old WT mice and RAD6B ncKO mice, each group containing 8 mice. All values are presented as the mean ± SEM (*n* = 8). Student’s *t*-test.

FIGURE S2 More neurodegeneration was observed in the brains of RAD6B-deficient mice. **(A)** Immunofluorescence was performed with anti-NeuN in the brain slices from RAD6B-deficient mice and control mice that were treated with 10Gy of X-rays or not. Scale bar = 20 μm. **(B)** The scatter diagram displays the relative density of neurons in the cerebral cortex of mice from each group. All values are presented as the mean ± SEM (*n* = 18). Student’s *t*-test. **(C)** Degeneration of nerve fibers (tangling, thickening, and staining) was observed in the silver-stained brain slices from RAD6B-deficient mice. Scale bar = 10 μm. **(D)** In lipofuscin staining of brain slices from RAD6B-deficient mice, microglia clustered around degenerated neurons. Scale bar = 10 μm. **(E)** The scatter diagram displays the proportion of abnormal neurons. All values are presented as the mean ± SEM (*n* = 18). Student’s *t*-test.
