# Supplementary figures and images for: RAD6B Plays a Critical Role in Neuronal DNA Damage Response to Resist Neurodegeneration
Source: Front Cell Neurosci. 2019 Aug 23;13:392. doi: 10.3389/fncel.2019.00392 (PMC6716356; doi:10.3389/fncel.2019.00392)

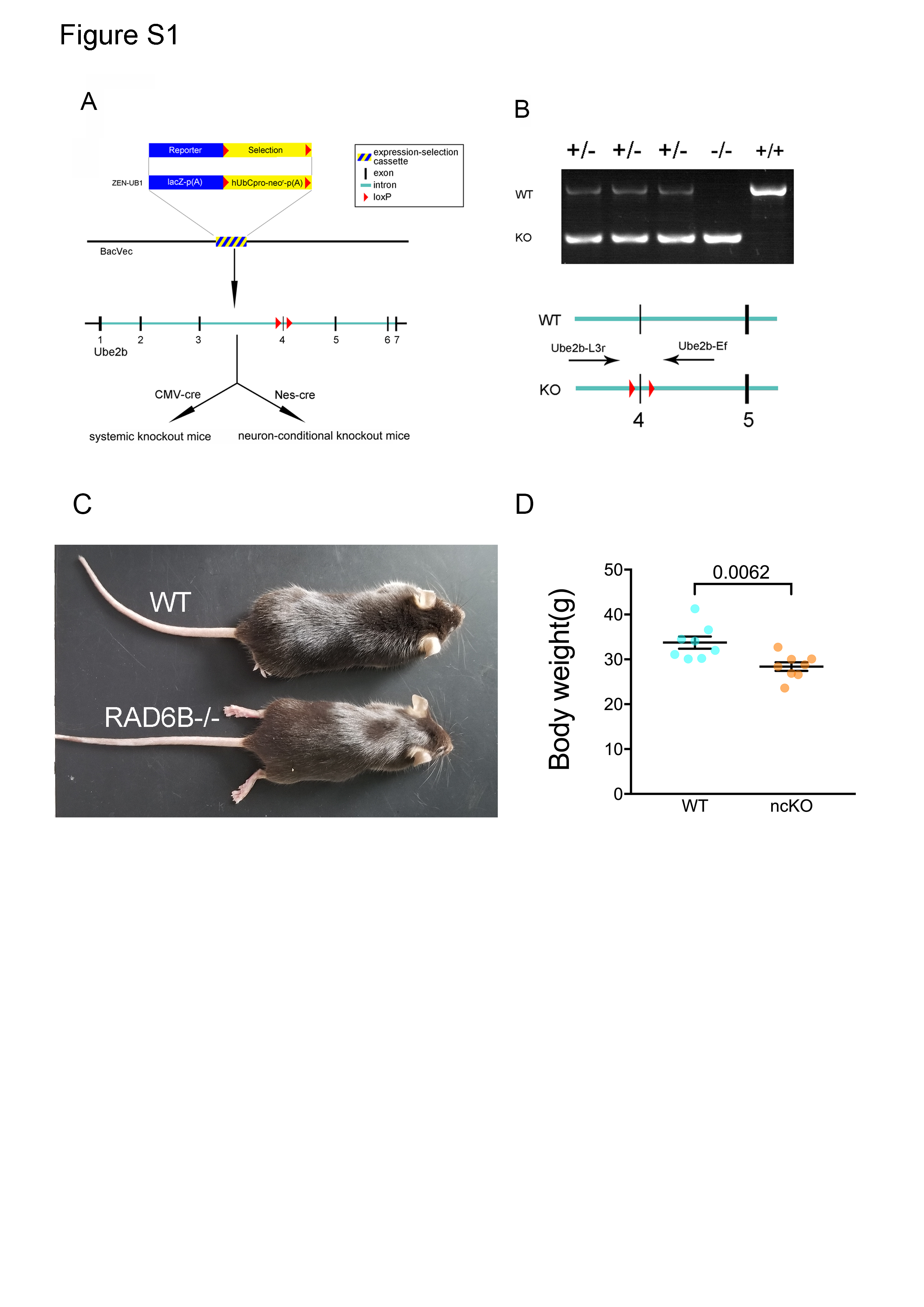

Supplement: FIGURE S1 — Implementation of RAD6B-deficient mice. (A) Editing the RAD6B gene through the Cre-loxP recombination system. CMV-cre and Nes-cre were used to produce systemic knockout mice and ncKO mice, respectively. (B) Identification of the genotypes of some offspring mice obtained from RAD6B heterozygous mice. Primers were designed for genome amplification. (C) Physical comparison of 4-month-old WT mice and RAD6B ncKO mice. (D) The scatter diagram shows weight differences between 18-month-old WT mice and RAD6B ncKO mice, each group containing 8 mice. All values are presented as the mean ± SEM (n = 8). Student’s t-test. [file Image_1.TIF]

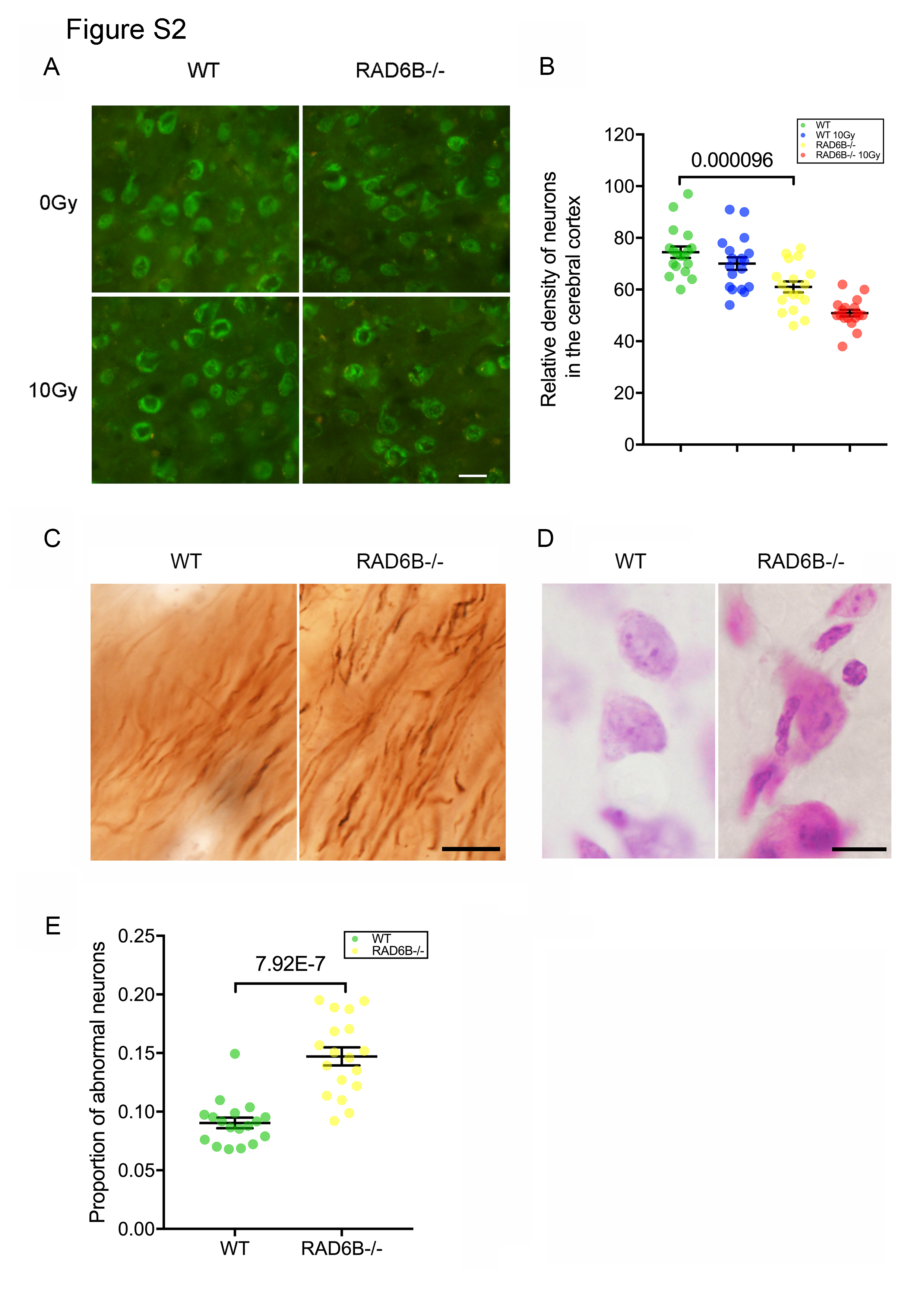

Supplement: FIGURE S2 — More neurodegeneration was observed in the brains of RAD6B-deficient mice. (A) Immunofluorescence was performed with anti-NeuN in the brain slices from RAD6B-deficient mice and control mice that were treated with 10Gy of X-rays or not. Scale bar = 20 μm. (B) The scatter diagram displays the relative density of neurons in the cerebral cortex of mice from each group. All values are presented as the mean ± SEM (n = 18). Student’s t-test. (C) Degeneration of nerve fibers (tangling, thickening, and staining) was observed in the silver-stained brain slices from RAD6B-deficient mice. Scale bar = 10 μm. (D) In lipofuscin staining of brain slices from RAD6B-deficient mice, microglia clustered around degenerated neurons. Scale bar = 10 μm. (E) The scatter diagram displays the proportion of abnormal neurons. All values are presented as the mean ± SEM (n = 18). Student’s t-test. [file Image_2.TIF]
